# Gemcitabine Plus Docetaxel, Dacarbazine, Doxorubicin Combinations, or Doxorubicin Alone as First-Line Treatment for Advanced/Metastatic Leiomyosarcoma: A Retrospective Analysis at a Sarcoma Center

**DOI:** 10.3390/diseases13030079

**Published:** 2025-03-11

**Authors:** Ted Kim, Clara Hao, Minggui Pan, Kristen N. Ganjoo, Nam Q. Bui

**Affiliations:** Division of Oncology, Department of Medicine, Stanford University, Stanford, CA 94305, USAminggui@stanford.edu (M.P.); kganjoo@stanford.edu (K.N.G.)

**Keywords:** leiomyosarcoma, soft tissue sarcoma, chemotherapy, doxorubicin, progression-free survival, overall survival, retrospective study, cancer treatment, Kaplan–Meier, disease control rate

## Abstract

Background/Objectives: Locally advanced and metastatic leiomyosarcoma (LMS) is an aggressive cancer with limited treatment options. This single-institution, retrospective study evaluated the efficacy of first-line chemotherapy regimens in patients with advanced or metastatic LMS treated at Stanford Medical Center. Methods: Seventy-four patients with unresectable or metastatic LMS were deemed eligible and treated with first-line chemotherapy regimens, including gemcitabine plus docetaxel, dacarbazine, doxorubicin combinations (with evofosfamide or ifosfamide), and doxorubicin monotherapy. Progression-free survival (PFS), overall survival (OS), and disease control rate (DCR) were assessed using RECIST v1.1, with survival analyses performed using Kaplan–Meier and Cox proportional hazards methods. Results: The cohort consisted of 56 females (75.7%) and 18 males (24.3%), with a median age of 55.5 years. The majority (93.2%) had metastatic disease. The median PFS for the entire cohort was 4.9 months (range: 0.6–28.1 mo), and the median OS was 27.3 months (range: 1.9–140.2 mo). The doxorubicin combination (DC) group had the highest median PFS of 7.9 months (range: 0.6–15.8 mo). Doxorubicin alone had the highest median OS of 33.8 months (4.2–100.2 mo). Doxorubicin combinations demonstrated superior PFS in both uterine and non-uterine LMS subgroups. Conclusions: These findings reaffirm the efficacy of doxorubicin-based combination regimens as a first-line treatment for locally advanced and metastatic LMS, particularly in non-uterine LMS.

## 1. Introduction

Soft tissue sarcomas (STSs) are a rare and complex heterogeneous group of mesenchymal neoplasms with over 150 different histological subtypes, accounting for approximately 1% of all adult solid tumors [1]. They commonly arise from the extremities (59%), trunk (19%), and retroperitoneum (15%), with undifferentiated pleomorphic sarcoma, liposarcoma, leiomyosarcoma, and synovial sarcoma being the most common histopathological subtypes [2]. STSs have a propensity to metastasize to the lung, with higher grade STS having a metastatic potential as high as 60% [2]. STS with metastatic disease has an overall survival between 12 and 19 months [2].

Leiomyosarcoma (LMS) is one of the most common STS subtypes, accounting for up to 25% of all newly diagnosed sarcomas. LMS is a smooth muscle tumor that can occur in both male and female patients, although it is more commonly found in females due to potential uterine origin [3]. They most commonly occur within the retroperitoneum, uterus, extremities, and trunk, with incidence increasing with age and peaking in the seventh decade of life [4]. The most recent 2020 World Health Organization (WHO) Classification of Soft Tissue and Bone Tumours stratifies smooth muscle tumors as either benign, intermediate, or malignant, and now includes the addition of distinct entities of both EBV-associated smooth muscle tumors and inflammatory leiomyosarcoma. Leiomyosarcoma and inflammatory leiomyosarcoma are the only two smooth muscle tumors to be classified as malignant, as EBV-associated smooth muscle tumors and smooth muscle tumors of uncertain malignant potential are classified as intermediate. Leiomyomas are classified as benign smooth muscle tumors [5,6].

Histologically, LMS originates from smooth muscle cells typically characterized by spindle-shaped cells in intersecting fascicles with elongated nuclei. Varying degrees of pleomorphism can occur, with variants of LMS that include pleomorphic, myxoid, and undifferentiated LMS. Grossly, they can appear as solitary and well-defined lesions that may have areas of cystic degeneration and necrosis. They are primarily associated with *RB1* and *PTEN* mutations involved in tumor suppression. Immunohistochemistry is positive for smooth muscle-specific markers including muscle-specific actin, desmin, and h-caldesmon [4]. Differential diagnoses can include leiomyomas, malignant peripheral nerve sheath tumors (MPNSTs), perivascular endothelial cell tumors (PEComas), synovial sarcoma, and undifferentiated pleomorphic sarcomas (UPSs), highlighting the importance of pathological review from established sarcoma centers or from pathologists with experience in diagnosing sarcomas.

Histologic grade, tumor size, and tumor depth remain the major clinicopathological prognostic factors for LMS and are staged according to the American Joint Committee of Cancer (AJCC) staging system. Uterine LMS (uLMS), however, has been associated with a worse prognosis and is staged according to the Federation of Gynecology and Obstetrics (FIGO) staging system [7]. Surgical resection with negative margins, with or without radiation therapy, is the gold standard for localized, resectable disease [3]. For uLMS, hysterectomy is recommended for patients with disease limited to the uterus only [7]. However, first-line treatment for recurrent advanced or metastatic LMS is limited to conventional chemotherapy, with doxorubicin monotherapy or combination gemcitabine/docetaxel remaining the standard chemotherapeutic treatment for STS for decades. Efficacy studies of first-line treatment for metastatic LMS with doxorubicin monotherapy have demonstrated a median progression-free survival (PFS) of 4.8 months and median overall survival (OS) of approximately 30 months [3].

Our observational study aimed to assess the outcomes of patients with advanced LMS treated with various chemotherapy regimens in the first-line setting at Stanford Medical Center. These regimens included doxorubicin alone, doxorubicin plus ifosfamide, doxorubicin plus dacarbazine, gemcitabine plus docetaxel, or more recently, doxorubicin plus trabectedin. Additionally, we aimed to stratify our findings between uterine and non-uterine LMS cohorts based on the unique characteristics and prognostic differences between the two entities, to assess differences in survival characteristics based on the type of first-line chemotherapy regimen given. Our results provide additional insight into understanding the efficacy of various regimens in LMS and may help guide treatment selections in patients with advanced LMS with special consideration to whether they are of uterine or non-uterine origin.

## 2. Materials and Methods

### 2.1. Patient Selection

This was a single institution retrospective observational study conducted at Stanford Medical Center and approved by the institutional review board. Eligible patients must have had locally advanced (unresectable) or metastatic leiomyosarcoma confirmed by histopathology at Stanford and have been treated with at least 1 cycle in the first-line setting with chemotherapy. The choice of first-line chemotherapy was based on physician discretion based on factors such as patient performance status, comorbidities, and disease burden, in alignment with the standard of care therapies and institutional treatment practices at the time. Patients must have had measurable disease, as per the Response Evaluation Criteria in Solid Tumors (RECIST) v1.1, and an age ≥ 18 years.

### 2.2. Data Collection

Data for this study were extracted from the institutional electronic medical records database at Stanford Medical Center. Eligible patients were identified and filtered based on the predefined inclusion and exclusion criteria. Relevant clinical data, including patient demographics, histopathological confirmation of leiomyosarcoma, treatment regimens, and disease progression, were collected. Patients were then grouped according to the specific first-line chemotherapy regimen they received.

### 2.3. Outcomes

The main purpose of this study was to retrospectively evaluate the efficacy of different first-line chemotherapeutic regimens for the treatment of advanced recurrent/metastatic leiomyosarcoma at Stanford Medical Center. Primary endpoints included progression-free survival (PFS), overall survival (OS), and disease control rate (DCR) per the Response Evaluation Criteria in Solid Tumors (RECISTv1.1). PFS was defined as the time from the start of treatment to disease progression or censorship due to treatment discontinuation secondary to toxicity or the initiation of a new therapy, whichever occurred first. OS was defined as the time from the start of treatment to death from any cause or censorship due to loss to follow-up. DCR was defined as the proportion of patients who achieved complete response (CR), partial response (PR), or stable disease (SD) as their best response to treatment, according to the RECISTv1.1, relative to the total number of evaluable patients.

### 2.4. Statistical Analysis

Categorical and continuous variables were assessed with percentages and means; no formal statistical hypothesis testing was performed with these variables. Progression-free survival (PFS) and overall survival (OS) were estimated using the Kaplan–Meier method and were compared between groups using the log-rank test. A Cox proportional hazards model was used for multivariate analysis. All analyses were performed using R version 4.2 and Python version 3.9.

## 3. Results

### 3.1. Demographics

**Figure 1:**
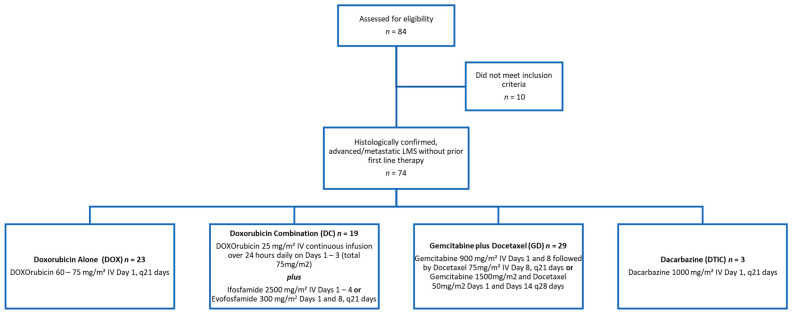
Patient enrollment and drug schedule.

Seventy-four patients with unresectable or metastatic LMS treated in the first-line setting at Stanford Medical Center were deemed eligible and were included in the study (Figure 1). This cohort of leiomyosarcoma patients (LMS, *n* = 74) consisted of 56 females (75.7%) and 18 males (24.3%), with more patients diagnosed with a primary site of extrauterine LMS (*n* = 50) than uterine LMS (*n* = 22). Patients were between 23 and 78 years of age, with a median age of 55.5 years old. At the treatment start, sixty-nine patients had metastatic disease (93.2%) and five patients had locally advanced disease not amenable to surgery (6.8%). The chemotherapy regimens that were analyzed consisted of doxorubicin alone (DOX, *n* = 23, 31.1%), (which includes doxorubicin plus olaratumab as trials showed no significant difference with the addition of olaratumab); doxorubicin combinations (DC, *n* = 19, 25.7%), which include doxorubicin plus evofosfamide (*n* = 16, 21.6%) and doxorubicin plus ifosfamide (*n* = 3, 4.1%); gemcitabine plus docetaxel (GD, *n* = 29, 39.2%); and dacarbazine (DTIC, *n* = 3, 4.1%) (Table 1). The best overall response across all treatment groups consisted of partial response (PR) of 5.4%, stable disease (SD) of 62.2%, and progression of disease (PD) of 32.4%.

### 3.2. Survival Outcomes

**Figure 2:**
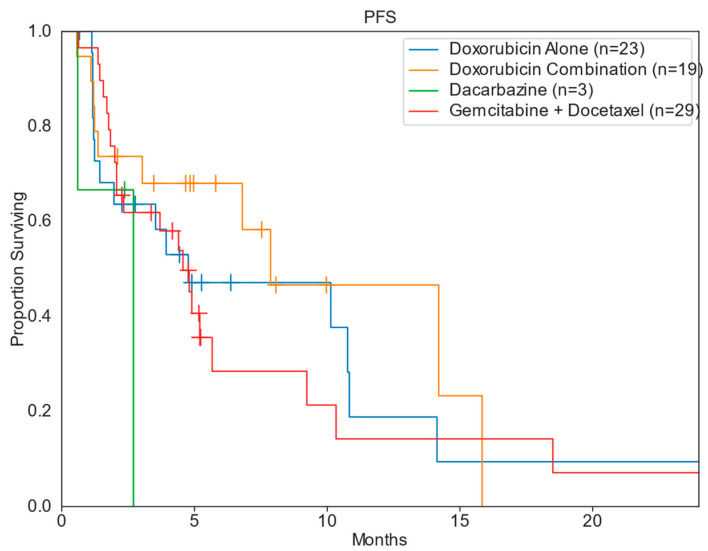
Kaplan–Meier progression-free survival.

**Figure 3:**
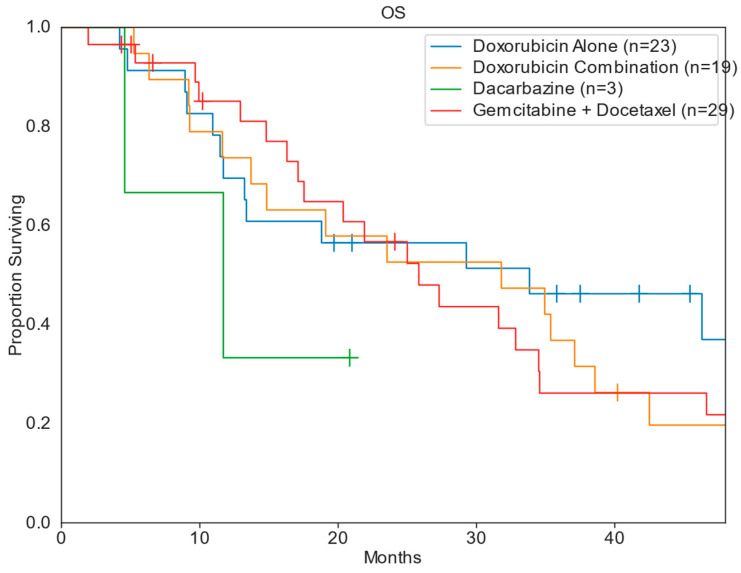
Kaplan–Meier overall survival.

**Figure 4:**
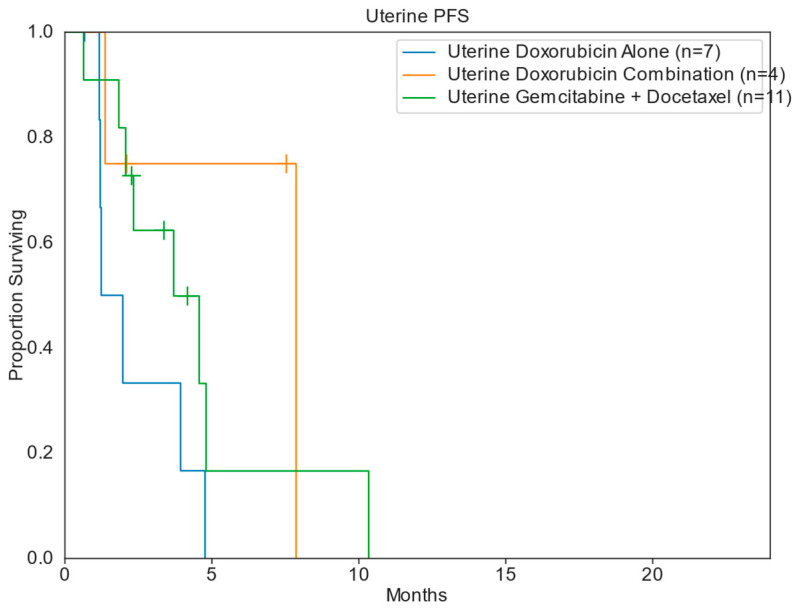
Kaplan–Meier uterine progression-free survival.

**Figure 5:**
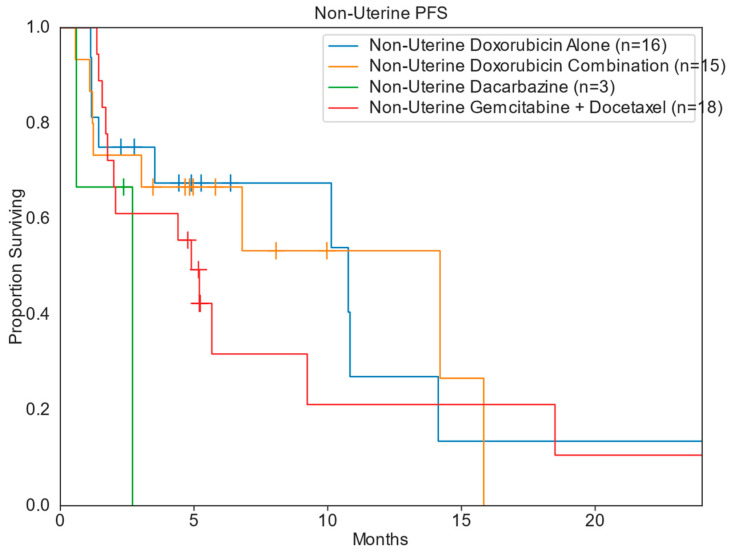
Kaplan–Meier non-uterine progression-free survival.

The median PFS and OS for patients across all treatment groups was 4.9 months and 27.3 months, respectively. Patients treated in the DC cohort had the greatest median PFS of 7.9 months (range: 0.6–15.8 mo), compared to DOX [4.8 months (range: 0.7–28.1 mo)], GD [4.6 months (range: 0.6–25.5 mo)], and DTIC [2.7 months (range: 0.6–2.7 mo)]. The DOX cohort had the greatest median OS of 33.8 months, compared to 31.8 months for DC, 25.8 months for GD, and 2.7 months for DTIC (Table 2; Figure 2 and Figure 3). When stratifying for uterine primary tumors, DC had a median PFS of 7.9 months, compared to 3.7 months for GD and 2.0 months for DOX (Table 3; Figure 4). Nonuterine primary PFS for DC and DOX were 14.2 and 10.8 months, respectively, while GD and DTIC were much lower at 4.9 and 2.7 months, respectively (Table 4; Figure 5).

## 4. Discussion

This study evaluated patients diagnosed with metastatic or advanced/unresectable LMS at a single sarcoma center, who were treated with different regimens of first-line chemotherapy to evaluate PFS and OS data in STS. The results from this study further support previous studies showing the efficacy of doxorubicin combinatorial regimens, doxorubicin alone, and gemcitabine plus docetaxel in leiomyosarcoma. This study was also able to segregate a specific population of only LMS patients to evaluate these chemotherapy regimens in this unique histological subtype and further characterize treatment response depending on the location of the primary site of disease (uterine vs. non-uterine). The overall PFS and OS data are consistent with those from more recent clinical studies in STS and LMS. The doxorubicin combination had the highest median PFS across all LMS, uterine and non-uterine. Notably, doxorubicin alone seemed to be less effective in the treatment of uterine LMS, with only a PFS of less than 2 months, while a combination of doxorubicin chemotherapy with another agent resulted in a far greater median PFS of 14.2 months. This is particularly important since uterine LMS has been shown to have a worse OS compared to non-uterine LMS [8]. Interestingly, GD combination, although a common option in the treatment of STS, had inferior results in PFS in LMS overall compared to doxorubicin combinations and doxorubicin alone, as well as in non-uterine LMS, while only having a slightly greater PFS than the doxorubicin-only treatment group in uterine LMS (3.7 vs. 2.0 months). Of course, these hypotheses could be influenced by the small sample size of subgroup analyses.

A phase 3 randomized controlled trial (EORTC 62012) was conducted at 38 hospitals across 10 countries studying the efficacy of doxorubicin alone versus intensified doxorubicin plus ifosfamide for first-line treatment of advanced or metastatic STS. This phase 3 study found that the PFS was significantly higher in the intensified doxorubicin plus ifosfamide group compared to the doxorubicin alone group (7.4 months vs. 4.6 months), although there was no improvement in the OS when comparing both groups, with the study authors concluding that the results did not support the use of intensified doxorubicin plus ifosfamide for STS except in the case of the specific goal of tumor shrinkage prior to surgical resection. Although there was no significant difference in OS when comparing both groups in the study, it is important to note that the response rate was found to be higher in the doxorubicin plus ifosfamide group compared to doxorubicin alone (26% vs. 14%), thus patients with high disease burden or those that are very symptomatic and need immediate cytoreduction would benefit from combination therapy. Additionally, grade 3 and 4 toxic effects, including leukopenia, neutropenia, febrile neutropenia, anemia, and thrombocytopenia, were found to be more common with doxorubicin and ifosfamide than with doxorubicin alone [9]. Based on these findings, the benefits of doxorubicin combination therapy in tumor reduction and cytoreduction, despite the increased relevant toxicities, must be considered along with patient characteristics such as tumor burden and performance status when making treatment decisions.

The retrospective study conducted by D’Ambrosio et al. (2020) then compared doxorubicin plus ifosfamide versus doxorubicin plus dacarbazine or doxorubicin alone as first-line treatments for advanced LMS. The results from their study of more than 300 patients with advanced or metastatic LMS showed that doxorubicin plus dacarbazine had a more favorable PFS and OS compared to doxorubicin plus ifosfamide, while the cohort treated with doxorubicin alone had the least favorable median PFS and OS in their analyses [3].

Following multiple phase 2 studies supporting the activity and efficacy of gemcitabine–docetaxel in STS, a randomized controlled phase 3 study was conducted in 2017 comparing gemcitabine–docetaxel to the gold standard of doxorubicin alone in previously untreated advanced unresectable or metastatic STS [10,11,12]. A total of 257 patients were enrolled and subsequently randomly assigned to the two different treatment arms, gemcitabine–docetaxel, or doxorubicin. At the conclusion of the study, analyses revealed that the median PFS was similar across both arms (23.3 weeks vs. 23.7 weeks). OS also did not significantly differ between the two groups; the 24-week overall survival rate was 82.6% in the gemcitabine and docetaxel group and 82.6% in the doxorubicin group. The median OS was 76.3 weeks in the doxorubicin group and 67.3 weeks in the gemcitabine and docetaxel group. Interestingly, the investigators conducted further subgroup analyses looking at efficacy data for both groups separated out by histopathological diagnoses but did not find any differential effect or evidence between the two groups for leiomyosarcomas or any other STS subtypes [11]. However, more studies specifically recruiting recurrent advanced and metastatic LMS may warrant further investigation into the potential treatment response in this subtype of STS.

More recently, trabectedin has demonstrated some level of efficacy in STS with an objective response rate of 10% in patients that have failed previous doxorubicin plus ifosfamide [13]. Trabectedin has even shown further benefit in patients with pretreated LMS and liposarcoma compared to other STS histological subtypes, with a reported 6-month PFS of 26–30% [14,15,16]. Since then, other studies have investigated doxorubicin combined with trabectedin as a potential therapy and have shown promising results in phase 1 and 2 studies in the treatment of LMS [17,18,19,20]. From these studies, the randomized, multicenter open-label phase 3 trial (LMS-04) compared the PFS of patients with metastatic or unresectable LMS treated with doxorubicin plus trabectedin as first-line therapy versus doxorubicin alone. The median PFS was significantly longer with doxorubicin plus trabectedin versus doxorubicin alone (12.2 months [95% CI 10.1–15.6] vs. 6.2 months [4.1–7.1], further demonstrating the efficacy of this combination. However, the combination therapy resulted in a higher, but manageable, toxicity profile [16]. More recent data published from this trial now reports a longer median OS in the doxorubicin plus trabectedin group (33 months) compared to the doxorubicin-only group (24 months). PFS findings were consistent with previous reports from the study, with doxorubicin plus trabectedin resulting in a longer median PFS of 12 months compared to just 6 months in the doxorubicin-only group [21].

This retrospective study offered a unique opportunity to evaluate the efficacy of common STS chemotherapeutic regimens in a very specific cohort of untreated metastatic or advanced/recurrent LMS in the first-line setting. Nevertheless, the study presents some limitations; being a single-center retrospective study with a relatively small sample size, the results from this study can potentially not be representative of a larger population, and treatment decisions are biased by institutional preferences. Furthermore, safety data were not collected in this study as differing chemotherapy regimens and combinatorial chemotherapies can often have higher toxicity profiles, which may impact the decision to place patients into specific treatment lines based on patient functional status. Higher toxicities may have also resulted in premature discontinuation of the study drug prior to reaching a disease endpoint.

## 5. Conclusions

In summary, our study shows that the treatment of metastatic or advanced LMS with doxorubicin combinations in the first-line setting led to a favorable PFS compared to other common lines of first-line therapies even when stratifying between uterine and nonuterine primary sites, potentially helping guide treatment decisions in this specific patient population. Future directions in research for the treatment of metastatic or advanced LMS could include looking into optimizing chemotherapy regimens utilizing other drugs that are known to be active in LMS. Further elucidating molecular mechanisms that play pivotal roles in the proliferation of LMS could provide potential targets for targeted and immunotherapies, as well as new and emerging treatments utilizing antibody-drug conjugates and other novel cell therapies.

## Figures and Tables

**Table 1:** Baseline population characteristics.

	Doxorubicin Alone (*n* = 23)	Doxorubicin Combination (*n* = 19)	Gemcitabine + Docetaxel (*n* = 29)	Dacarbazine (*n* = 3)	Total (*n* = 74)
Age at Diagnosis, y					
Median (Range)	55 (35–73)	58 (23–75)	55 (28–78)	61 (32–72)	55.5 (23–78)
Sex, No. (%)					
Female Sex	21 (91.3)	13 (68.4)	20 (69.0)	2 (66.7)	56 (75.7)
Male Sex	2 (8.7)	6 (31.6)	9 (31.0)	1 (33.3)	18 (24.3)
Site of Primary Tumor, No. (%)					
Uterine	7 (30.4)	4 (21.1)	11 (37.9)	0 (0.0)	22 (29.7)
Extrauterine	16 (69.6)	14 (73.7)	17 (58.6)	3 (100.00)	50 (67.6)
Largest Tumor Size, mm					
Median (range)	50 (15–120)	64 (20–228)	61 (6–195)	42 (22–187)	55 (6–228)
Mean	52.6	78.0	73.0	83.7	68.4
Disease Status, No. (%)					
Locally Advanced	2 (8.7)	0 (0.0)	2 (6.9)	1 (33.3)	5 (6.8)
Metastatic	21 (91.3)	19 (100.0)	27 (93.1)	2 (66.7)	69 (93.2)

**Table 2:** Progression-free survival and overall survival.

	DOX (*n* = 23)	DC (*n* = 19)	GD (*n* = 29)	DTIC (*n* = 3)	Total (*n* = 74)
PFS, Median (Range), Mo	4.8 (0.7–28.1)	7.9 (0.6–15.8)	4.6 (0.6–25.5)	2.7 (0.6–2.7)	4.9 (0.6–28.1)
OS, Median (Range), Mo	33.8 (4.2–100.2)	31.8 (5.2–140.2)	25.8 (1.9–126.1)	11.7 (4.6–20.8)	27.3 (1.9–140.2)
Disease Control Rate, %	65.2	73.7	69.0	33.3	67.6

**Table 3:** Median uterine progression-free survival.

	PFS, Median (Range), mo
Doxorubicin Combination	7.9 (1.4–7.9)
Dox Only	2.0 (0.7–4.8)
GD	3.7 (0.6–10.3)

**Table 4:** Median non-uterine progression-free survival.

	PFS, Median (Range), mo
Doxorubicin Combination	14.2 (0.6–15.8)
Dox Only	10.8 (1.1–28.1)
GD	4.9 (1.4–25.5)
DTIC	2.7 (0.6–2.7)

## Data Availability

The data presented in this study are available on request from the corresponding author.
